# Cellular HIV-1 DNA levels in patients receiving antiretroviral therapy strongly correlate with therapy initiation timing but not with therapy duration

**DOI:** 10.1186/1471-2334-11-146

**Published:** 2011-05-24

**Authors:** Dai Watanabe, Shiro Ibe, Tomoko Uehira, Rumi Minami, Atsushi Sasakawa, Keishiro Yajima, Hitoshi Yonemoto, Hiroki Bando, Yoshihiko Ogawa, Tomohiro Taniguchi, Daisuke Kasai, Yasuharu Nishida, Masahiro Yamamoto, Tsuguhiro Kaneda, Takuma Shirasaka

**Affiliations:** 1 AIDS Medical Center, National Hospital Organization Osaka National Hospital, Osaka, Japan; 2 Clinical Research Center, National Hospital Organization Nagoya Medical Center, Nagoya, Japan; 3 Internal Medicine, Clinical Research Institute, National Hospital Organization Kyushu Medical Center, Fukuoka, Japan; 4 College of Pharmacy, Kinjo Gakuin University, Nagoya, Japan

## Abstract

**Background:**

Viral reservoir size refers to cellular human immunodeficiency virus-1 (HIV-1) DNA levels in CD4^+ ^T lymphocytes of peripheral blood obtained from patients with plasma HIV-1-RNA levels (viral load, VL) maintained below the detection limit by antiretroviral therapy (ART). We measured HIV-1 DNA levels in CD4^+ ^lymphocytes in such patients to investigate their clinical significance.

**Methods:**

CD4^+ ^T lymphocytes were isolated from the peripheral blood of 61 patients with a VL maintained at less than 50 copies/ml for at least 4 months by ART and total DNA was purified. HIV-1 DNA was quantified by nested PCR to calculate the copy number per 1 million CD4^+ ^lymphocytes (relative amount) and the copy number in 1 ml of blood (absolute amount). For statistical analysis, the Spearman rank or Wilcoxon signed-rank test was used, with a significance level of 5%.

**Results:**

CD4 cell counts at the time of sampling negatively correlated with the relative amount of HIV-1 DNA (median = 33 copies/million CD4^+ ^lymphocytes; interquartile range [IQR] = 7-123 copies/million CD4^+ ^lymphocytes), but were not correlated with the absolute amounts (median = 17 copies/ml; IQR = 5-67 copies/ml). Both absolute and relative amounts of HIV-1 DNA were significantly lower in six patients in whom ART was initiated before positive seroconversion than in 55 patients in whom ART was initiated in the chronic phase, as shown by Western blotting. CD4 cell counts before ART introduction were also negatively correlated with both the relative and absolute amounts of HIV-1 DNA. Only the relative amounts of HIV-1 DNA negatively correlated with the duration of VL maintenance below the detection limit, while the absolute amounts were not significantly correlated with this period.

**Conclusions:**

The amounts of cellular HIV-1 DNA in patients with VLs maintained below the detection limit by the introduction of ART correlated with the timing of ART initiation but not with the duration of ART. In addition, CD4^+ ^T lymphocytes, which were newly generated by ART, diluted latently infected cells, indicating that measurements of the relative amounts of cellular HIV-1 DNA might be underestimated.

## Background

Anti-human immunodeficiency virus (HIV) drugs can suppress viral replication but cannot directly eliminate latently HIV-1-infected cells. Replication-competent HIV-1 can persist in a stable latent reservoir in CD4^+ ^T lymphocytes and monocytes, thus carrying integrated HIV-1 DNA. Among these cells, resting memory CD4^+ ^T lymphocytes constitute a major latent reservoir [1]. Siliciano et al. calculated the number of latently infected cells as the frequency of replication-competent virus per 1 million CD4^+ ^lymphocytes in peripheral blood and reported that their half-life was about 44 months [2]. The number of latently infected cells in an HIV-1-infected patient's body is estimated to be approximately 1 million [3]. The report concluded that an HIV-infected patient's body undergoing anti-HIV therapy (ART) would take 73.4 years for complete viral elimination; thus, ART would need to be continued for the rest of the patient's life [4]. However, the method for calculating the frequency of replication-competent virus in this previous report is not necessarily sensitive enough. An alternative method for estimating the viral reservoir size of a patient receiving ART is to quantify cellular HIV-1 DNA in infected cells.

Many studies have reported the amount of cellular HIV-1 DNA in peripheral blood. In particular, the recent use of real-time PCR has allowed the straightforward and accurate measurement of HIV-1 DNA. It also enables us to distinguish all forms of intracellular HIV-1 DNA, including integrated and unintegrated linear DNA, as well as 1-long terminal repeat (LTR) and 2-LTR circles. The total HIV-1 DNA in peripheral blood mononuclear cells (PBMC) and in CD4^+ ^lymphocytes after prolonged viral suppression largely corresponds to integrated HIV-1 DNA [5,6]. This evidence indicates that integrated HIV-1 DNA is the most stable form in patients receiving ART, and that viral reservoir sizes can therefore be estimated by examining the amounts of cellular HIV-1 DNA.

We reported the detection limit of real-time PCR, using the LightCycler system, to be 500 copies per 1 million cells in peripheral blood; therefore, HIV-1 DNA in 30% of the patients receiving ART could not be quantified using the conventional real-time PCR method [7]. In a subsequent study, we improved the detection level of HIV-1 DNA levels with real-time PCR by including a pre-amplification step in the first PCR, followed by quantification with a second PCR [8]. Specifically, we PCR-amplified the β2-microglobulin (β2 M) gene and HIV-1 DNA simultaneously in the same tube, quantified the products by TaqMan PCR, and then determined the amounts of HIV-1 DNA using the copy number of amplified HIV-1 DNA and the amplification efficiency of β2 M. This method improved the detection limit to 2 copies/10^6 ^cells. Here, we measured the amount of HIV-1 DNA in CD4^+ ^lymphocytes in the peripheral blood using this novel, highly sensitive method in HIV patients who have undergone ART for a prolonged period, and in whom the plasma HIV-1 RNA levels (viral load, VL) remained undetectable.

## Methods

### Patients and study design

Adult patients visiting either the Osaka National Hospital or the Kyushu Medical Center and whose VL levels remained below the detection limit (50 copies/ml) for 4 months or longer were included in this cross-sectional analysis of an open-labeled cohort of HIV-1-infected patients successfully treated with ART. Written informed consent for collection of peripheral blood was obtained from 69 patients. Of these patients, 8 patients with a history of rebound of VLs (>400 copies/ml) were excluded from the study. This study was reviewed and approved by the institutional review boards of the Osaka National Hospital and relevant institutions.

### Estimation of the number of CD4+ T lymphocytes (CD4 cell count) and HIV-1 VL

CD4 cell counts were measured by flow cytometry using the whole-blood lysis method. VLs were measured using the reverse transcription PCR method (AMPLICOR HIV-1 monitor test, Roche Molecular Diagnostic), with a detection limit of 50 copies/ml, according to the manufacturer's instructions. Serum anti-HIV-1 antibody levels were detected using LAV Blot I (Bio-Rad Laboratories). Sera were determined to be positive for the antibody according to the criteria of the World Health Organization.

### Isolation of CD4-positive T lymphocytes and DNA extraction

Peripheral blood was collected with an EDTA blood collecting tube. CD4-positive T lymphocytes were isolated from whole blood using StemSep column chromatography (Stem Cell Technologies). The collected cells were then washed with phosphate-buffered saline and resuspended. The purity of CD4-positive T lymphocytes was more than 98% by flow cytometry. For DNA extraction, 1-5 × 10^6 ^cells were used. DNA was extracted using the QIAamp DNA Blood Mini Kit (QIAGEN) according to the manufacturer's instructions.

### Quantification of HIV-1 DNA

HIV-1 DNA was quantified by real-time PCR as previously reported [8]. A second round of PCR was conducted using the extracted DNA as a template. First, the human β2 M gene and HIV-1 DNA (*Gag*) were simultaneously amplified in the same tubes used for pre-quantification PCR. The first round of PCR consisted of 20 cycles of amplification. Subsequently, TaqMan PCR was used to determine the copy numbers of the pre-amplified human β2 M gene and HIV-1 DNA. The copy number of HIV-1 DNA was calculated as the copy number per 10^6 ^cells (relative amount) using the amplification efficiency of human β2 M. The copy number of HIV-1 DNA contained in CD4^+ ^T lymphocytes in 1 ml of blood was determined as an absolute amount. The absolute amount of HIV-1 DNA was calculated using the following formula:

### Statistical analysis

For statistical analysis, the Wilcoxon signed-rank test was used for intergroup comparisons and the Spearman rank test was used for correlation analysis. All analyses were conducted with a significance level of 5%.

## Results

**Figure 1:**
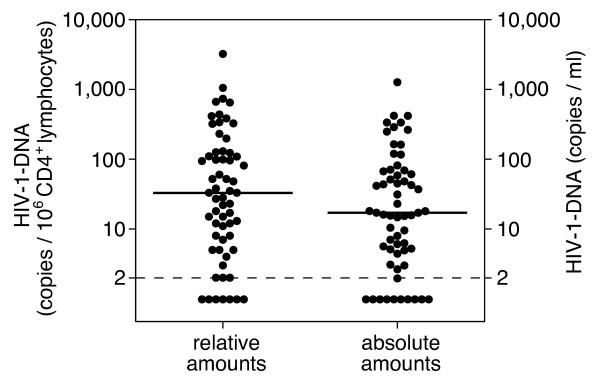
**Relative and absolute HIV-1 DNA levels in all patients**. The dots indicate the amounts of intracellular HIV-1 DNA per 10^6 ^CD4^+ ^lymphocytes (relative amount, left) and the amounts of HIV-1 DNA per 1 ml of blood (absolute amount, right). The dotted line indicates the detection limit for HIV-1 DNA.

The general characteristics of the 61 patients included in the study are presented in Table 1. CD4^+ ^lymphocytes were isolated from peripheral blood, and HIV-1 DNA contained in these isolated cells was quantified using the method described above. The distributions of values for relative and absolute amounts of HIV-1 DNA in CD4^+ ^lymphocytes are shown in Figure 1. The absolute amounts were generally lower than the relative ones. However, both relative and absolute amounts exhibited a similar distribution. Seven patients (11%) had values below the detection limit (2 copies/10^6 ^CD4^+ ^lymphocytes) when relative amounts were assessed, whereas 10 patients (16%) had values below the detection limit (2 copies/ml) when absolute amounts were assessed.

**Table 1:** Demographic and clinical characteristics of HIV-1-infected patients

Characteristic		Absolute value	
Number of participants		61	
Number of male participants, (%)		56	(92%)
Age (y), median [IQR]		43	[35-48]
Route of transmission, (%)			
	homosexual	46	(76%)
	heterosexual	13	(21%)
	blood products	2	(3%)
Nationality, (%)			
	Japanese	61	(100%)
Nadir CD4 cell count (/μl), median [IQR]*		175	[54-256]
Pre-ART VL (copies/ml), median [IQR]*		85500	[41500-322500]
Current CD4 cell count (/μl), median [IQR]		569	[442-818]
Current ART regimen, no. (%)			
	PI-based	32	(52%)
	NNRTI-based	25	(41%)
	3-NRTI	4	(7%)
Duration of VL suppression (days), median [IQR]		2205	[784-2737]

* Data are unavailable for seven (11%) patients. IQR, interquartile range; VL, viral load; ART, antiretroviral therapy; PI, protease inhibitor; NNRTI, non-nucleoside reverse transcriptase inhibitor; NRTI, nucleoside reverse transcriptase inhibitor.

**Figure 2:**
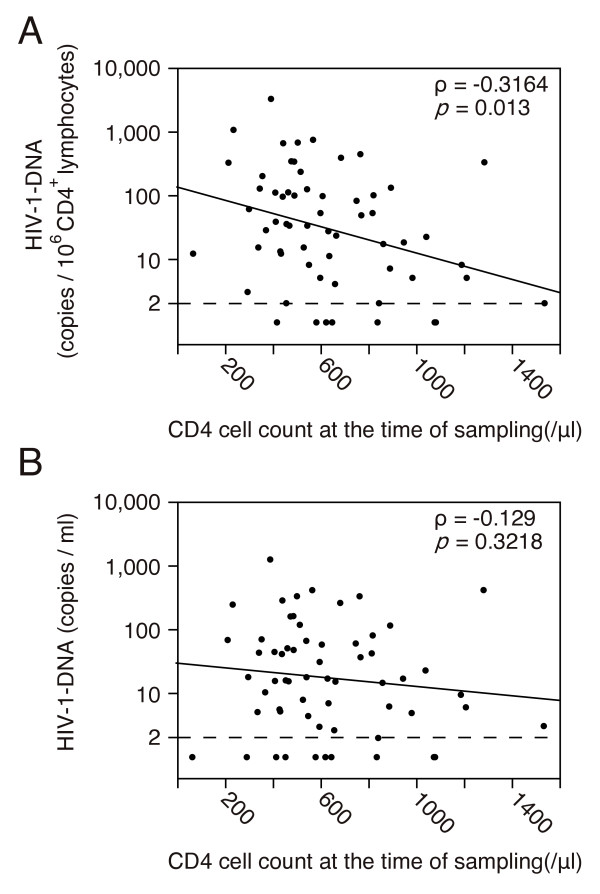
**Correlation between HIV-1 DNA levels and CD4 cell counts at the time of sampling**. (A) Negative correlation between the relative amounts and CD4 cell counts at the time of sampling. Spearman's rank test was used for statistical analysis. Spearman's correlation coefficients (ρ) and p-values are indicated in the figure. The straight line (y = 2.12 - 0.00103x, p = 0.01) was calculated using linear regression analysis. Samples below the detection limit were assumed to be 1 copy in the calculation. (B) There was no correlation between the absolute amounts and CD4 cell counts at the time of sampling. Spearman's rank test and linear regression analysis (y = 1.47 - 0.000368x, p = 0.3342) were used for statistical analysis.

The correlation between the amounts of HIV-1 DNA and CD4 cell counts at the time of sampling was examined using Spearman's rank test (Figure 2). A significant negative correlation was noted between the relative amounts of HIV-1 DNA and CD4 cell counts (Spearman's ρ = -0.3164, p = 0.013). This indicates that the latently infected cells may be diluted with newly generated T lymphocytes after the introduction of ART. However, there was no significant correlation between the absolute amounts of HIV-1 DNA calculated as copy numbers in 1 ml of blood and CD4 cell counts at this time point (Figure 2B).

**Figure 3:**
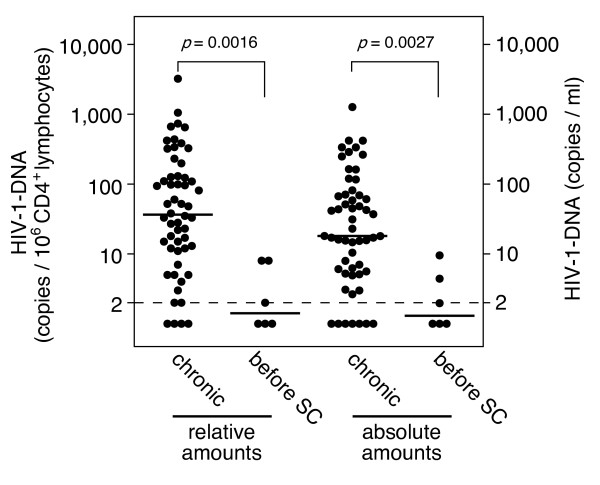
**HIV-1 DNA levels in patients in whom the therapy was introduced before seroconversion**. The relative (left) and absolute amounts (right) of HIV-1 DNA in patients in whom therapy was initiated during the chronic phase (chronic) or before seroconversion (before SC). The Wilcoxon test was used for statistical analysis.

**Figure 4:**
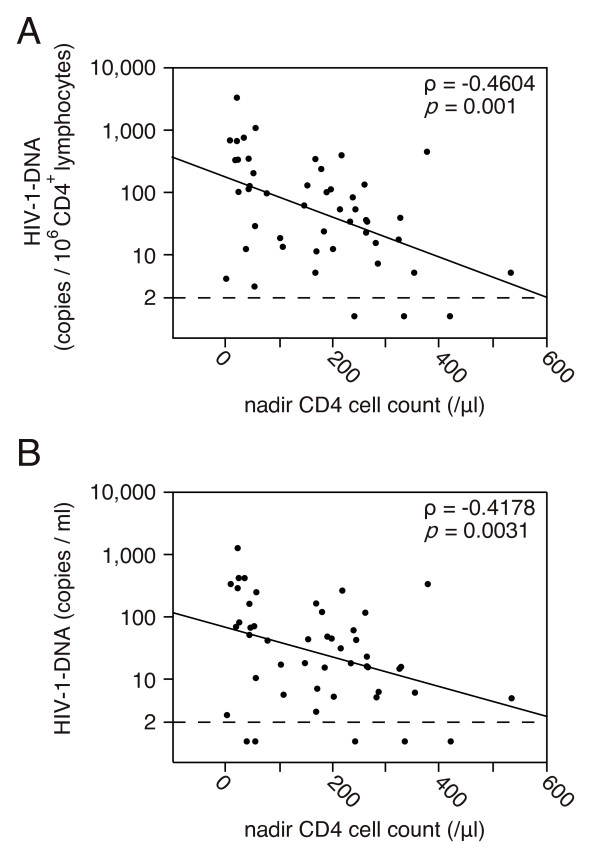
**Correlation between HIV-1 DNA levels and CD4 cell counts before ART introduction**. (A) Relative amounts (best-fit line: y = 2.23 - 0.00321x, p = 0.0005) and (B) absolute amounts (best-fit line: y = 1.83 - 0.00237x, p = 0.0085) of HIV-1 DNA levels.

The patients were divided into 2 groups depending on when ART was introduced: 6 patients in whom ART was introduced before positive seroconversion, as shown by Western blotting (before-SC group), and 55 patients in whom ART was introduced in the chronic phase (chronic group). The two groups were compared to determine the association between the amounts of HIV-1 DNA and the timing of ART introduction (Figure 3). In the before-SC group, for which therapy was initiated before seroconversion, HIV-1 DNA was not detected in half of the patients (three patients) (i.e., below the detection limit of 2 copies/10^6 ^CD4^+ ^lymphocytes). In the chronic group, HIV-1 DNA was not detected in 4 of 55 patients (7%). Comparison of the amounts of HIV-1 DNA between these two groups demonstrated that the relative and absolute amounts were lower in the before-SC group than in the chronic group (p = 0.016 and p = 0.0027, respectively). Subsequently, we examined the correlation between the CD4 cell counts immediately before the initiation of ART (nadir CD4 cell counts) and the amounts of HIV-1 DNA. We analyzed nadir CD4 cell counts in 48 patients in whom ART was initiated in the chronic phase; nadir CD4 cell counts in 7 patients were unavailable at the time of the study. As shown in Figure 4, both the relative and absolute amounts negatively correlated with nadir CD4 cell counts. No significant correlation was noted between the amounts of HIV-1 DNA and the VLs immediately before the introduction of ART (relative: Spearman's ρ = 0.2192, p = 0.1344; absolute: Spearman's ρ = 0.2471, p = 0.0904). Collectively, these observations indicate that the amounts of cellular HIV-1 DNA in HIV-1-infected patients during ART correlated with the time when therapy was initiated. Moreover, the amounts of cellular HIV-1 DNA were maintained at lower levels, particularly in patients in whom ART was initiated earlier after HIV-1 infection.

We next investigated the association between the amount of HIV-1 DNA and the current ART regimen. The 61 patients were divided into 3 groups according to the antiretroviral drugs they received: protease inhibitor-based regimen (PI group, n = 32), non-nucleoside reverse transcriptase inhibitor-based regimen (NNRTI group, n = 25), and 3-nucleoside reverse transcriptase inhibitor regimen (3NRTI group, n = 4). Amongst the three groups, the HIV-1 DNA levels were highest in the 3NRTI group. However, no significant differences were observed in either the relative or absolute amounts of HIV-1 DNA among the three groups (Wilcoxon signed-rank test, relative: p = 0.3325, absolute: p = 0.4091). Median HIV-1 DNA levels in the PI group (relative: 50 copies/10^6 ^CD4^+ ^lymphocytes; absolute: 25 copies/ml) were similar to those in the NNRTI group (relative: 23 copies/10^6 ^CD4^+ ^lymphocytes; absolute: 17 copies/ml).

**Figure 5:**
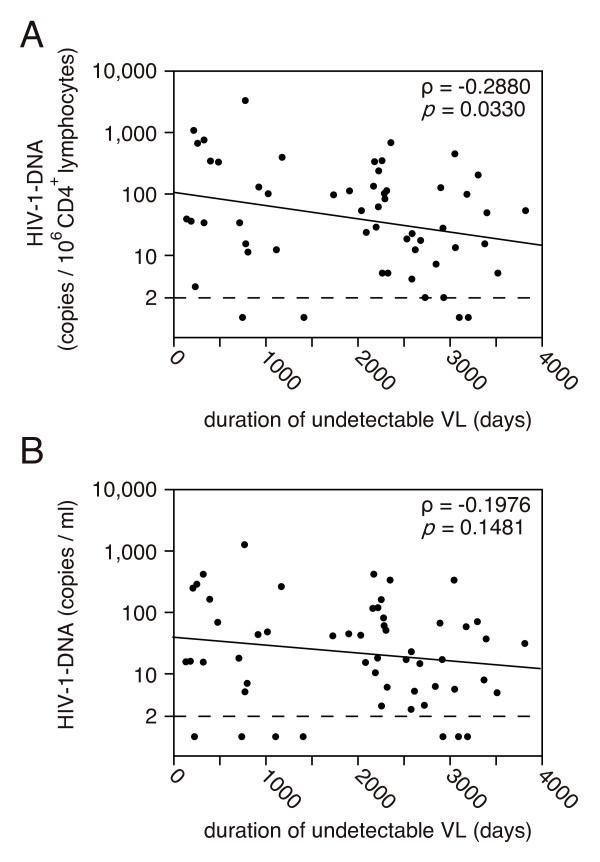
**Correlation between the amounts of HIV-1 DNA and the period with undetectable VL**. (A) Relative amounts (best-fit line: y = 2.02 - 0.000216x, p = 0.0459), and (B) absolute amounts (best-fit line: y = 1.60 - 0.000128x, p = 0.2147) of HIV-1 DNA levels.

Finally, the amounts of HIV-1 DNA and the duration of ART were examined. The duration of ART was defined as the period during which VL was maintained below the detection limit. The relative amounts of HIV-1 DNA showed a significant negative correlation with the duration of undetectable VLs (Figure 5A). A regression analysis, conducted based on the assumption that the amounts of HIV-1 DNA in patients with their VLs below the detection limit were 1 copy/10^6 ^CD4^+ ^lymphocytes, provided a straight line, as shown in Figure 5A (p = 0.0459). The half-life of HIV-1 DNA calculated from the regression line was about 1,400 days (47 months). As shown in Figure 5B, there was no correlation between the absolute amounts of HIV-1 DNA and the duration of undetectable VLs. In addition, the half-life of HIV-1 DNA was extended to about 2,300 days (78 months; p = 0.215). The duration of ART and the CD4 cell counts at the time of sampling were moderately correlated (data not shown, Spearman's ρ = 0.400, p = 0.001), suggesting that the CD4 cell counts increased as the duration of ART increased.

## Discussion

In this study, the amounts of cellular HIV-1 DNA in peripheral CD4^+ ^lymphocytes were measured in patients with VLs continuously maintained by ART below the detection limit using a novel, highly sensitive method [8]. Collectively, the data presented here indicate that while the viral replication was favorably suppressed with anti-HIV drugs for a prolonged period, HIV-1 DNA can be quantified in about 90% of these patients. This is similar to the detection limits previously reported [9,10].

In addition to the nested PCR methods, quantifying HIV-1 DNA in isolated CD4^+ ^lymphocytes contributed to improved sensitivity of the assay because these cells are the major reservoir of PBMC, and their use increases the population containing HIV-1 DNA [1]. However, purification of CD4^+ ^lymphocytes eliminates minor viral reservoir cells, such as monocytes, from the analysis. The amounts of HIV-1 DNA in monocytes in patients with HIV-1-associated dementia are higher than those in HIV-1-infected patients without dementia [11,12]. Furthermore, ultra-deep pyrosequencing has shown that there is high HIV-1 heterogeneity in monocytes compared with CD4^+ ^lymphocytes, suggesting a possible source of viral diversity [13]. Whilst these observations highlight the importance of examining monocyte reservoirs, we used only the CD4+ lymphocyte population rather than PBMC to improve the sensitivity of cellular HIV-1 DNA detection in this study.

The amount of cellular HIV-1 DNA in CD4^+ ^lymphocytes is generally calculated as the copy number per 1 million CD4^+ ^lymphocytes or per 1 μg of total DNA. In this study, the values calculated using this method were defined as the relative amounts. As reported previously, a negative correlation was noted between the relative amounts of HIV-1 DNA and CD4 cell counts at the time of sampling [6,14], and the duration of therapy [15-18]. These results indicate that HIV-1-uninfected CD4^+ ^lymphocytes may be newly generated during ART, which could dilute the amounts of latently infected cells. Thus, an examination of the relative amounts may result in an underestimation of the amounts of HIV-1 DNA, and may not correctly reflect the viral reservoir of an HIV-1-infected patient's body. The relative amounts were corrected for the CD4 cell counts at the time of sampling, and were also analyzed as copy number in CD4^+ ^lymphocytes in 1 ml of blood (absolute amounts). After this correction, there was no negative correlation between the amounts of HIV-1 DNA and CD4 cell counts at the time of sampling. Thus, it seemed appropriate to examine absolute amounts as well as relative amounts of HIV-1 DNA in CD4^+ ^lymphocytes to determine the effect of ART on viral reservoir size.

A negative correlation between the relative amounts of HIV-1 DNA and the duration of ART was noted in this study. A regression analysis demonstrated that the amounts of cellular HIV-1 DNA were halved when VLs were suppressed below the detection limit for about 47 months. This half-life was almost equivalent to that of latently infected cells previously reported by Siliciano et al. (about 44 months) [2]. In this previous report, the half-life was calculated by counting latently infected cells per 1 million CD4^+ ^lymphocytes for 7 years, and the researchers concluded that it took 73.4 years to eliminate all latently infected cells. However, the possible dilution of latently infected cells with newly generated CD4^+ ^lymphocytes was not addressed. As indicated in this study, there was no correlation between the absolute amounts and duration of therapy, and the half-life was extended to about 78 months. Although the accuracy of the half-life calculated here is limited by the fact that this study is not a longitudinal study, previous reports of the time required for ART to eliminate HIV-1 from patients' bodies are likely to be underestimates.

In addition to the decline rate of latently infected cells in the CD4^+ ^lymphocyte reservoir, the decline rate of cellular HIV-1 DNA in PBMC has also been investigated. Longitudinal studies involving patients undergoing long-term ART have shown that the amount of cellular HIV-1 DNA in PBMC decreased by 2.5-3.0 copies/10^6 ^PBMC in the first year within initiating ART [16,18,19]; this was followed by a milder decrease and levels reached a plateau after about 80 weeks [20,21] or 3 years [18]. These results resemble the decline in the absolute amounts of cellular HIV-1 DNA rather than the relative amounts seen in the present study, although the comparison between the amounts of HIV-1 DNA in PBMC and relative and absolute amounts of HIV-1 DNA in CD4^+ ^lymphocytes was not performed in this study.

No differences were observed in the amount of HIV-1 DNA between the PI and NNRTI groups in this study. In a previous observational study, the amounts of HIV-1 DNA in the PI and NNRTI groups showed no significant differences using the Kruskal-Wallis test [22]. However, in this previous study, subjects with high levels of HIV-1 DNA were more common in the NNRTI group than in the PI group, suggesting the possibility that PI treatment may have a greater impact on viral reservoir than NNRTI. These two previous studies, however, did not conclusively address the effects of anti-HIV drugs on viral reservoir because they were not randomized controlled studies. Nevertheless, the use of drugs that exert a beneficial effect on the clearance of HIV-1 DNA might reduce the half-life of latently infected cells. Recently, Buzón et al. reported that intensification of ART treatment with raltegravir, an HIV-1 integrase inhibitor, in patients on standard ART led to a transient increase in 2-LTR circles [23]. This suggests that ongoing viral replication persists despite suppressive ART, and that raltegravir prevents linear HIV-1 DNA from integrating into chromatin, followed by conversion of linear DNA to 2-LTR circles. The continual replenishment of viral reservoirs by ongoing viral replication is thought to be a mechanism responsible for the longevity of viral reservoir. Further studies are required to determine whether raltegravir can reduce the half-life of the viral reservoir by arresting ongoing viral replication that seems to occur during ART.

In agreement with previous reports [24,25], this study demonstrates that the amounts of HIV-1 DNA are maintained at low levels in patients in whom ART was initiated before the production of anti-HIV antibody. Previous reports that examined the impact of 1 year of ART showed that ART reduces the cellular HIV-1 DNA level more effectively when initiated during the acute rather than the chronic phase of HIV-1 infection. Given these observations, our results indicate that this effect could continue for a prolonged period because our patients received long-term ART (median = approximately 6 years). It should be noted that six patients in whom therapy was initiated before seroconversion required the introduction of ART to alleviate the symptoms of acute HIV infection. Most of these patients had more severe symptoms than patients with usual acute HIV infection. Thus, it is unlikely that the amounts of HIV-1 DNA were maintained at low levels by low viral replication. In addition to these observations, the CD4 cell counts before the introduction of ART were negatively correlated with the amounts of HIV-1 DNA. Pre-ART VLs also tended to correlate with the amounts of HIV-1 DNA, but without statistical significance. This suggests that the latently infected cells might be established early after infection [26,27] and increase gradually during an asymptomatic period. In support of this, several studies have followed asymptomatic carriers with stable VLs for a long time and shown a gradual increase in cellular HIV-1 DNA [28] and an increase in the amount of HIV-1 DNA together with the progression of immunodeficiency [29,30].

The amounts of HIV-1 DNA were significantly lower, in patients in whom ART was introduced before seroconversion. Because HIV-1 DNA could not be detected in three of the six patients of the before-SC group, this study cannot demonstrate the actual suppression level of cellular HIV-1 DNA in these patients. Moreover, these results need to be interpreted carefully in light of some conflicting results on the amount of HIV-1 DNA in patients treated with ART during the acute phase [31,32], and negative results about the potential clinical usefulness of estimation of the HIV-1 DNA level [33-36]. The present study is limited by its small cohort size (especially the before-SC group), and blood samples were obtained from patients enrolled at two centers, resulting in a cross-sectional study design. Therefore, further longitudinal studies are required to confirm our preliminary findings from a small cohort of patients. In the future, we hope to develop a more sensitive analysis method to determine the effects of ART intervention in the acute phase of HIV-1 infection by examining the relationship between the amounts of cellular HIV-1 DNA and long-term course of ART.

## Conclusions

Here, we analyzed samples from 61 patients to examine the significance of the amounts of HIV-1 DNA. The amounts of HIV-1 DNA were correlated with the timing of ART introduction, but not with the duration of ART. In addition, CD4^+ ^T lymphocytes, newly generated by ART, diluted latently infected cells, indicating that the relative amounts of cellular HIV-1 DNA might be underestimated. In addition to analysis of the relative amount, quantifying the absolute amount of cellular HIV-1 DNA would be helpful in providing an accurate measure of HIV-1 DNA in CD4^+ ^T lymphocytes.

## Competing interests

The authors declare that they have no competing interests.

## Authors' contributions

TK and TS participated in the study design and coordination; SI and DW carried out the experiments; TS, TU, DW, RM, AS, KY, HY, HB, YO, TT, DK, YN, and MY managed the patients and collected the data; DW and YN wrote the paper. All authors read and approved the final manuscript.

## Pre-publication history

The pre-publication history for this paper can be accessed here:

http://www.biomedcentral.com/1471-2334/11/146/prepub
